# Valsartan in Combination with Tripterygium Glycosides Protects against Chronic Nephritis via the Toll-Like Receptor 4 Pathway

**DOI:** 10.1155/2022/4807028

**Published:** 2022-08-24

**Authors:** Jiabao Dong, Duo Huang, Ling Jing, Mengmeng Wu

**Affiliations:** Maanshan Shiqiye Hospital, 828 Hunan West Road, Yushan District, Maanshan, 243000 Anhui, China

## Abstract

**Objective:**

Valsartan has been studied to exert effects on kidney disease. However, the concrete function of valsartan in combination with tripterygium glycosides in chronic nephritis remained largely unknown. The study was designed to unravel the impacts of valsartan and tripterygium glycosides in chronic nephritis through the Toll-like Receptor 4 (TLR4) pathway.

**Methods:**

The renal function indicators such as serum creatinine (Scr), blood urea nitrogen (BUN) and *β*2 microglobulin (*β*2-MG), 24 h urine protein **(**Upro) levels, and blood lipid indicators such as total cholesterol (TC), low-density lipoprotein (LDL-C), triacylglycerol (TG) and high-density lipoprotein (HDL-C), inflammatory factors (e.g., IL-1*β* and IL-8), and the proportion of T lymphocyte subpopulations (CD4+ and CD8+) were detected in chronic nephritis patients before and after treatment with valsartan alone or valsartan combined with tripterygium glycosides. Symptoms of adverse reactions were recorded. TLR4 expression in the patients' serum was examined.

**Results:**

Compared to patients before treatment, after treatment with valsartan alone or valsartan combined with tripterygium glycosides, the renal function indicators Scr, BUN, and 24 h levels were reduced, and TC, TG, and LDL-C levels were reduced, while HDL-C levels were elevated; inflammatory responses (IL-1*β* and IL-8) were mitigated; CD4+ ratio and CD4+/CD8+ ratio increased yet CD8+ ratio decreased; TLR4 expression was silenced after treatment. All of the changes were more obvious in patients after being treated with valsartan combined with tripterygium glycosides.

**Conclusion:**

Valsartan in combination with tripterygium glycosides protects against chronic nephritis via suppressing the Toll-like Receptor 4 pathway.

## 1. Introduction

Glomerulonephritis is relatively a rare kidney disease with substantial morbidity and mortality [1]. Chronic glomerulonephritis, also known as chronic nephritis, may first present as chronic kidney disease of any stage. Patients with chronic glomerulonephritis are typically also affected by haematuria, proteinuria, and hypertension, and it may be detected by renal ultrasound [2]. Immunoglobulin A nephropathy (IgAN) is a major common cause of chronic glomerulonephritis globally [3]. Most glomerulonephritides frequently afflict young people, often cannot be thoroughly cured, causing multiple chronic kidney diseases, such as end-stage renal failure, with associated high morbidity and enormous cost [4]. Considering the challenge of chronic nephritis treatment, it is necessitated to further explore novel therapeutic targets for chronic nephritis.

As is an orally active antihypertensive drug developed in the last century, valsartan is a selective angiotensin II receptor blocker 1 which relieves the blood vessels and thus reduces blood pressure [5]. Angiotensin II blockade has been acknowledged a standard antifibrotic therapeutic modality in kidney disease. It has been reported that the optimal dose of valsartan contributes to exerting the antifibrotic effect by protecting podocytes in glomerulonephritis [6]. Moreover, valsartan plus activated vitamin D has better therapeutic effect than valsartan alone in mitigating the moderate proteinuria in IgA nephropathy and without additional adverse events [7]. As for the combination therapy, valsartan with tripterygium glycosides from Tripterygium wilfordii Hook F can reduce proteinuria in patients with diabetic nephropathy [8]. Tripterygium glycoside is a traditional Chinese medicine. A previous research has revealed that Tripterygium wilfordii polyglycosides can weaken the inflammatory state of chronic nephritis [9]. In addition, it has been unveiled that tripterygium glycoside tablet contributes to ameliorating renal tubulointerstitial fibrosis through the Toll-Like Receptor 4 (TLR4) pathway [10]. TLR4 in HBV-GN was higher in hepatitis B virus-associated glomerulonephritis [11]. Specifically, the deficiency of TLR4 can modulate the levels of inflammatory factors in IgA nephropathy [12]. As stated above, valsartan and tripterygium glycosides play vital roles in the development of kidney-related diseases. Enlightened by previous findings, the study was implemented to unravel the combined effects of valsartan and tripterygium glycosides in chronic nephritis via the TLR4 signaling pathway, thus affording effective modalities for chronic nephritis treatment.

## 2. Materials and Methods

### 2.1. Ethics Statement

The study was ratified by the Ethics Committee of Maanshan Shiqiye Hospital. All the participants have signed the written consent.

### 2.2. Study Subjects

From November 2018 to January 2020, 110 patients with chronic nephritis admitted to Maanshan Shiqiye Hospital were classified into a control group (treated with valsartan, *n* = 55) and an observation group (treated with valsartan and tripterygium glycosides, *n* = 55).

Inclusion criteria for patients were displayed as follows: (1) aged 28-65 years, (2) glomerulonephritis by various clinical examinations, (3)24 h urine protein (24 h Upro) more than 3.5 g and plasma albumin less than 30 g/L, and (4) signed the informed consent form.

Exclusion criteria for patients were listed as follows: (1) acute infection, (2) a history of immunosuppressant use in the two weeks prior to enrollment, (3) immune system or hematologic disorders, (4) patients with malignancy or severe cardiovascular disease, and (5) patients with hypersensitivity to the drug used in the study [9].

### 2.3. Grouping and Treatment

Patients with chronic nephritis in the control group were given valsartan (160 mg/day) alone, once a day; in the observation group, patients were further given tripterygium glycosides (Lunan Houpu Pharmaceutical Co., Ltd., Shandong, China, Z37020344, specification: 10 mg/tablet), 20 mg (2 tablets)/time orally, three times a day. Patients in both groups were treated continuously for 2 months [13].

### 2.4. Renal Function Index

Patients in the control and observation groups had fasting venous blood harvested before and two months after treatment, and the venous blood was centrifuged to separate the serum. Serum creatinine (Scr), blood urea nitrogen (BUN), serum *β*2 microglobulin (*β*2-MG), and other renal function indices were measured with an automatic biochemical analyzer (Hitachi, Tokyo, Japan) [14].

### 2.5. Urine Protein (Upro) Level Measurement

A 24-hour urine volume was collected and a fully automated biochemical analyzer was used to measure and compare the 24 h Upro levels pre- and posttreatment between the two groups.

### 2.6. Blood Lipid Index

Venous blood was harvested from both groups before and two months after treatment, and total cholesterol (TC), low-density lipoprotein (LDL-C), triacylglycerol (TG), and high-density lipoprotein (HDL-C) were tested using an automated biochemical analyzer [15].

### 2.7. Inflammatory Factor Assessment

Blood samples were collected, and the levels of cytokines interleukin- (IL-) 1*β* and IL-8 were tested using ELISA kits (Sigma, St. Louis, MO, USA) [16].

### 2.8. Flow Cytometry

Collected venous blood was utilized for the assessment of the ratio of T-lymphocyte subpopulations (CD4+ and CD8+). The CD4+/CD8+ ratio was calculated by the flow cytometer (BD Biosciences, NJ, USA) and its adjuvant reagents.

### 2.9. Detection of Incidence of Adverse Reactions

Adverse reactions were recorded in both groups during the treatment. Incidence of adverse reactions was calculated with the number of adverse reactions/total number of cases ×100% [9].

### 2.10. RT-qPCR

Fasting venous blood was obtained before and two months after treatment. The supernatant was collected by centrifugation. Fasting venous blood was collected from healthy controls as above. Total RNAs were extracted using TRIzol reagent and reversely transcribed to complementary DNAs with a SuperScript™ III reverse transcriptase. An ABI PRISM® 7000 Sequence Detection System together with the SYBR® Green PCR Master Mix kit was adopted for quantitative real-time PCR reactions. Primer sequences are described in Table 1. The mRNA expression was evaluated with the 2^-*ΔΔ*CT^ method, and then, relative mRNA expression was normalized to GAPDH [17].

### 2.11. Statistical Analysis

All statistical analyses were processed using SPSS 21.0 software and GraphPad Prism 6. Data conformed to normal distribution was calculated with the Kolmogorov-Smirnov test. The measurement data were represented as means ± standard deviation. Comparisons between the two groups were examined by the *t*-test. Count data were expressed as number of cases *n* (%). The *χ*^2^ test or Fisher's exact test was used. Statistical significance was determined when *P* < 0.05.

## 3. Results

### 3.1. Clinical Data

As listed in Table 2, the difference in the baseline data was not significant between the two groups (all *P* > 0.05).

### 3.2. Valsartan in Combination with Tripterygium Glycosides Improves the Renal Function of Chronic Nephritis Patients

As suggested in Table 3, before treatment, the differences in Scr, BUN, and *β*2-MG levels between the two groups presented no statistical significant (all *P* > 0.05). After treatment, there exhibited lower levels of Scr, BUN, and *β*2-MG in both groups in comparison to before treatment, and lower levels of these factors were seen in the observation group versus the control group (all *P* < 0.05).

### 3.3. Valsartan in Combination with Tripterygium Glycosides Reduces 24 h Upro Levels of Chronic Nephritis Patients

**Figure 1:**
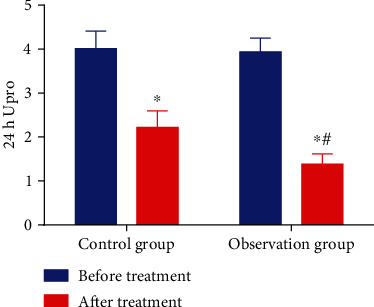
Valsartan in combination with tripterygium glycosides reduces 24 h Upro levels of chronic nephritis patients. ^∗^*P* < 0.05 vs. before treatment; ^#^*P* < 0.05 vs. the control group.

As reflected in Figure 1, the pretreatment 24 h Upro level was 3.94 ± 0.31 g and 4.01 ± 0.40 g in the observation and control groups, respectively. After treatment, the 24 h Upro level was 1.38 ± 0.24 g and 2.22 ± 0.38 g in the observation and control groups, respectively. It was observed that the 24 h Upro level was reduced in both groups after treatment and also lowered in the observation group versus the control group (*P* < 0.05).

### 3.4. Valsartan in Combination with Tripterygium Glycosides Reduces TC, LDL-C, and TG Levels yet Increases HDL-C Levels in Chronic Nephritis Patients

As indicated in Table 4, before treatment, there exhibited no differences in TC, LDL-C, TG, and HDL-C levels between the two groups (all *P* > 0.05). Compared with before treatment, TC, LDL-C, and TG levels were reduced, and HDL-C level was increased in both groups after treatment (all *P* < 0.05). After treatment, the levels of TC, LDL-C, and TG were lower, and the level of HDL-C was higher in the observation group than those in the control group (all *P* < 0.05).

### 3.5. Valsartan in Combination with Tripterygium Glycosides Attenuates the Inflammatory Response of Chronic Nephritis Patients

As shown in Table 5, before treatment, serum IL-1*β* and IL-8 levels harbored no change in the two groups (both *P* > 0.05). After treatment, a lower serum IL-1*β* and IL-8 levels were observed in both groups versus before treatment, and a lower IL-1*β* and IL-8 levels were presented in the observation group versus the control group (all *P* < 0.05).

### 3.6. Valsartan in Combination with Tripterygium Glycosides Elevates the CD4+ Ratio and CD4+/CD8+ Ratio while Reduces the CD8+ Ratio of Chronic Nephritis Patients

As suggested in Table 6, before treatment, the ratio of CD4+ or CD8+ and the ratio of CD4+/CD8+ exhibited no change between the two groups (all *P* > 0.05). After treatment, an elevation in both the CD4+ ratio and CD4+/CD8+ ratio and a reduction in the CD8+ ratio were witnessed in both groups. The change was more significant in the observation group (both *P* < 0.05).

### 3.7. The Adverse Reactions after the Treatment of Valsartan in Combination with Tripterygium Glycosides in Chronic Nephritis Patients

In order to investigate the symptoms of adverse reactions of chronic nephritis after the treatment of valsartan and tripterygium glycosides, we recorded the adverse reactions in the two groups, and the total incidence of adverse reactions in the two groups was compared in Table 7 (*P* > 0.05).

### 3.8. Valsartan in Combination with Tripterygium Glycosides Reduces TLR4 Levels of Chronic Nephritis Patients

**Figure 2:**
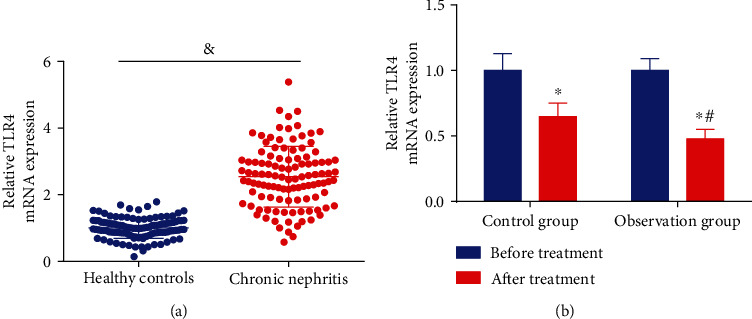
Valsartan in combination with tripterygium glycosides reduces TLR4 levels of chronic nephritis patients. (a) TLR4 levels in serum of healthy volunteers versus patients with nephritis were examined by RT-qPCR. (b) PCR to detect TLR4 expression in serum of patients before and after treatment in the observation and control groups was tested by RT-qPCR. ^&^*P* < 0.05 vs. healthy controls; ^∗^*P* < 0.05 vs. before treatment; ^#^*P* < 0.05 vs. the control group.

It has been reported that TLR4 is highly expressed in IgA1 nephropathy, and TLR4 may regulate IL-8 concentrations through the NF-*κ*B pathway to alleviate IgA nephropathy and that [12]. For further unraveling the role of TLR4 pathway in chronic nephritis, we first measured the expression of TLR4 in serum of 110 normal volunteers (healthy control) and 110 patients with chronic nephritis before treatment. The results unveiled that TLR4 expression was augmented in patients with chronic nephritis (Figure 2(a)). Thereafter, the expression of TLR4 was then measured by RT-qPCR in the serum of patients pre- and posttreatment in the observation and control groups. The results elucidated that TLR4 levels were reduced posttreatment, and the decrement in TLR4 expression was even more obvious in the observation group treated with combination drugs (Figure 2(b)).

## 4. Discussion

As a type of kidney disease, glomerulonephritis is featured by inflammation within the renal glomeruli and small blood vessels [18]. The study focused on the combined therapeutic effects of valsartan and tripterygium glycosides in chronic nephritis. Collectively, it was manifested that valsartan and tripterygium glycosides could protect against chronic nephritis via the TLR4 signaling pathway.

Initially, the chronic nephritis patients were given valsartan alone and valsartan combined with tripterygium glycosides, respectively. The results came out that valsartan in combination with tripterygium glycosides reduces 24 h Upro levels, mitigates inflammatory response, and improves the renal function of chronic nephritis patients. Previous studies for directly invested the role of valsartan and tripterygium glycosides chronic nephritis were rare, yet in renal-related diseases, such as IgAN, it has been uncovered that the combined treatment with valsartan and clopidogrel and leflunomide can attenuate the urinary proteins loss and renal function exacerbation for IgAN patients, which further causes minimal adverse reactions [19]. Similarly, it has been suggested that low-dose valsartan can effectively reduce proteinuria instead of causing any intolerability in normotensive IgAN patients [20]. As for the Upro levels, Xiaowei et al. have elucidated that the combined treatment of valsartan and activated vitamin D effectively contributes to reducing urinary protein excretion in patients with IgAN [7]. The combined treatment effects of valsartan and tripterygium glycosides have been evidenced by Lengnan et al., who have reflected that Tripterygium glycosides can reduce proteinuria in patients with diabetic nephropathy [8].

Thereafter, it was manifested that the combined treatment of valsartan and tripterygium glycosides can also reduce the levels of TLR4, which was relatively highly expressed in chronic nephritis. The similar elevated TLR4 expression trend has also been tested in IgA nephropathy [21]. In chronic ischemic renal damage and IgA nephropathy, TLR4 also exerts a high level, which was implicated in exacerbated inflammatory response [22]. Furthermore, TLR4 expression is positively related to tubulointerstitial injury caused by glomerulonephritis [23]. As for the impacts of valsartan and tripterygium glycosides on TLR4 expression, a related study has reported that valsartan prevents glycerol-stimulated acute kidney injury via reducing the TLR4 and NF-kappaB expression [24]. Furthermore, in heart failure, it has been evidenced that valsartan can mitigate the inflammatory response by reducing TLR4 expression [25].

In conclusion, the study has manifested that the combined therapy of valsartan and tripterygium glycosides can reduce 24 h Upro levels, mitigates inflammatory response, and improves the renal function of chronic nephritis patients by repressing TLR4 expression. By unraveling the therapeutic effects of valsartan and tripterygium glycosides in chronic nephritis by TLR4 signaling pathway, the study advanced the understanding of the therapeutic modalities of chronic nephritis. Therefore, valsartan and tripterygium glycosides might be used as promising therapeutic drugs for the prevention and treatment of chronic nephritis. However, the impacts of TLR4 expression in chronic nephritis development were not discussed in detail, which could be explored in future works.

## Figures and Tables

**Table 1:** Primer sequences used for q-PCR.

Gene	Sequence (5′→3′)
TLR4	F: 5′-TTTGGACAGTTTCCCACATTGA-3′
	R: 5′-AAGCATTCCCACCTTTGTTGG-3′
GAPDH	F: 5′-GGAAGGTGAAGGTCGGAGTCA-3′
	R: 5′-GTCATTGATGGCAACAATATCCACT-3′

Note: TLR4: Toll-like Receptor 4; GAPDH: glyceraldehyde-3-phosphate dehydrogenase.

**Table 2:** Baseline data of the two groups.

	Control group (*n* = 55)	Observation group (*n* = 55)	*P*
Gender			0.178
Male (*n* (%))	35 (31.82%)	27 (24.55%)	
Female (*n* (%))	20 (18.18%)	28 (25.45%)	
Age (years)	43.90 ± 5.10	45.10 ± 6.20	0.270
Course of disease (years)	4.15 ± 1.29	4.67 ± 1.99	0.107
Basic diseases			0.625
Diabetes (*n* (%))	3 (2.73%)	4 (3.64%)	
Hypertension (*n* (%))	5 (4.55%)	7 (6.36%)	
Hyperlipemia (*n* (%))	5 (4.55%)	3 (2.73%)	

**Table 3:** Levels of renal function indices tested before and after treatment in both groups.

Groups	Scr (*μ*mol/L)	BUN (mmol/L)	*β*2-MG (mg/L)
*Control group (n* = 55)			
Before treatment	96.51 ± 7.21	8.78 ± 1.71	0.12 ± 0.05
After treatment	85.66 ± 6.24^∗^	7.41 ± 1.02^∗^	0.08 ± 0.04^∗^
*Observation group (n* = 55)			
Before treatment	98.64 ± 10.28	9.16 ± 1.64	0.14 ± 0.06
After treatment	78.91 ± 6.88^∗^^#^	6.41 ± 1.37^∗^^#^	0.05 ± 0.01^∗^^#^

Note: ^∗^*P* < 0.05 vs. the before treatment; ^#^*P* < 0.05 vs. the control group; Scr: serum creatinine; BUN: blood urea nitrogen; *β*2-MG: *β*2 microglobulin.

**Table 4:** Levels of blood lipid indices tested before and after treatment in both groups.

Groups	TG (mmol/L)	TC (mmol/L)	HDL-C (mmol/L)	LDL-C (mmol/L)
*Control group (n* = 55)				
Before treatment	1.94 ± 0.96	4.83 ± 0.80	1.19 ± 0.27	3.06 ± 0.71
After treatment	1.42 ± 0.65^∗^	4.57 ± 0.55^∗^	1.35 ± 0.31^∗^	2.79 ± 0.61^∗^
*Observation group (n* = 55)				
Before treatment	1.95 ± 0.92	4.82 ± 0.77	1.21 ± 0.26	3.04 ± 0.84
After treatment	1.08 ± 0.42^∗^^#^	4.23 ± 0.45^∗^^#^	1.48 ± 0.28^∗^^#^	2.49 ± 0.62^∗^^#^

Note: ^∗^*P* < 0.05 vs. the before treatment; ^#^*P* < 0.05 vs. the control group; TG: triacylglycerol; TC: total cholesterol; LDL-C: low-density lipoprotein; HDL-C: high-density lipoprotein.

**Table 5:** Levels of inflammatory factors tested before and after treatment in both groups.

Groups	IL-1*β* (pg/mL)	IL-8 (ng/L)
*Control group (n* = 55)		
Before treatment	15.22 ± 1.21	3.99 ± 1.71
After treatment	8.55 ± 0.19^∗^	2.39 ± 0.81^∗^
*Observation group (n* = 55)		
Before treatment	14.97 ± 0.91	3.84 ± 1.44
After treatment	5.52 ± 0.18^∗^^#^	1.59 ± 0.72^∗^^#^

Note: ^∗^*P* < 0.05 vs. the before treatment; ^#^*P* < 0.05 vs. the control group; IL: interleukin.

**Table 6:** Percentage of T-lymphocyte subpopulations before and after treatment in both groups.

Groups	CD4+ (%)	CD8+ (%)	CD4+/CD8+
*Control group (n* = 55)			
Before treatment	30.64 ± 5.88	33.99 ± 4.71	0.91 ± 0.29
After treatment	32.98 ± 4.97^∗^	29.99 ± 4.01^∗^	1.28 ± 0.19^∗^
*Observation group (n* = 55)			
Before treatment	30.22 ± 5.21	34.64 ± 5.44	0.88 ± 0.25
After treatment	34.99 ± 4.45^∗^^#^	27.59 ± 4.02^∗^^#^	1.39 ± 0.35^∗^^#^

Note: ^∗^*P* < 0.05 vs. the before treatment; ^#^*P* < 0.05 vs. the control group.

**Table 7:** Incidence of adverse reactions during treatment in both groups.

Groups	Nausea and vomiting (*n* (%))	Dizziness and headache (*n* (%))	Skin rash (*n* (%))	Elevated transaminases (*n* (%))	Total incidence (*n* (%))
Control group (*n* = 55)	2 (3.64%)	2 (3.64%)	1 (1.82%)	1 (1.82%)	6 (10.91%)
Observation group (*n* = 55)	2 (3.64%)	1 (1.82%)	0 (0.00%)	1 (1.82%)	4 (7.27%)
*P*	0.808				

## Data Availability

The original contributions presented in the study are included in the article; further inquiries can be directed to the corresponding author.
